# Recovery of hand function through mental practice: A study protocol

**DOI:** 10.1186/1471-2377-6-39

**Published:** 2006-10-26

**Authors:** Magdalena Ietswaart, Marie Johnston, H Chris Dijkerman, Clare L Scott, Sara A Joice, Steven Hamilton, Ronald S MacWalter

**Affiliations:** 1 School of Psychology and Sport Sciences, Northumbria University, Northumberland Building, Newcastle upon Tyne NE1 8ST, UK; 2 Helmholtz Institute, Department of experimental Psychology, Utrecht University, Heidelberglaan 2, 3584 CS Utrecht, The Netherlands; 3 Health Psychology Research Group, School of Psychology, University of Aberdeen, UK; 4 Department of Medicine for the Elderly, Grampian University Hospital Trust, Aberdeen, UK; 5 Stroke Studies Centre, Department of Medicine, Ninewells Hospital and Medical School, Dundee DD1 9SY, UK

## Abstract

**Background:**

The study aims to assess the therapeutic benefits of motor imagery training in stroke patients with persistent motor weakness. There is evidence to suggest that mental rehearsal of movement can produce effects normally attributed to practising the actual movements. Imagining hand movements could stimulate the redistribution of brain activity, which accompanies recovery of hand function, thus resulting in a reduced motor deficit.

**Methods/Design:**

A multi-centre randomised controlled trial recruiting individuals between one and six months post-stroke (n = 135). Patients are assessed before and after a four-week evaluation period. In this trial, 45 patients daily mentally rehearse movements with their affected arm under close supervision. Their recovery is compared to 45 patients who perform closely supervised non-motor mental rehearsal, and 45 patients who are not engaged in a training program. Motor imagery training effectiveness is evaluated using outcome measures of motor function, psychological processes, and level of disability.

**Discussion:**

The idea of enhancing motor recovery through the use of motor imagery rehabilitation techniques is important with potential implications for clinical practice. The techniques evaluated as part of this randomised controlled trial are informed by the current understanding in cognitive neuroscience and the trial is both of scientific and applied interest.

## Background

Stroke is a common and highly debilitating illness. Many patients (41–45%) experience chronic motor impairments [1] and limitations in activities of daily living [2] even after extensive neurological rehabilitation. They often result in long-term dependence at a considerable cost to the carers and the health service. It is therefore crucial to optimise motor recovery after stroke. In this manuscript we describe a novel protocol to increase motor recovery after stroke by using motor imagery.

Evidence for the idea that motor imagery training could enhance the recovery of hand function comes from several lines of research: the sports literature; neurophysiological evidence; health psychology research; as well as preliminary findings using motor imagery techniques in stroke patients.

Studies with healthy volunteers show that mental rehearsal can produce effects normally attributed to practising the actual movements [3-5] including an increase in muscle strength [6] and improved performance in highly-skilled athletes [7,8]. The latter finding suggests that motor imagery can increase performance even if used concurrent with intensive physical training. A recent study compared separate motor imagery training and physical training and found enhanced performance in both, compared to control groups [9].

Separate converging evidence comes from neurophysiological studies. Recovery of hand function after stroke is accompanied by a redistribution of activity within a network of parallel-acting multiple cortical motor areas [10,11]. Interestingly, imagining performing certain hand movements results in activation of several cerebral areas in which increased activity was observed after recovery of hand function [12,13]. Based on these observations we suggest that imagining hand movements could stimulate the redistribution of brain activity which accompanies recovery of hand function, thus resulting in a reduced motor deficit.

Physical practice has been shown to induce reorganisation of the areas adjacent to focal ischaemic infarction in the primary motor cortex in monkeys [14]. Furthermore, neuroimaging studies have demonstrated cortical functional reorganisation associated with recovery of hand function after three to four weeks of physical training in acute [15] and chronic stroke patients [16,17]. In addition, there is evidence for functional redistribution following motor imagery training in healthy volunteers, demonstrating that motor imagery training alone seems to be sufficient to promote the modulation of neural circuits leading to the same plastic changes in the motor system as those following repeated physical practice [18,19]. There is also some preliminary evidence for this in a stroke patient showing reorganisation within sensorimotor areas of the injured hemisphere following motor imagery training [20].

Evidence from the health psychology also provides support of the potential benefit of motor imagery training. Psychological processes in health and illness that play an influential role in determining recovery of motor functioning after stroke, such as perceived control [21,22] may also be mediated by engagement in a form of mental processing. Similarly, patients' ability to direct and sustain their attention is predictive of recovery in stroke patients [23] and may be enhanced by mental exercises such as motor imagery.

Several recent claims in the literature have noted the potential usefulness of mental practice using motor imagery in neurological rehabilitation [24-26], yet very little empirical work addresses the issue directly (for a systematic review see [27]). Some direct evidence for the benefits of motor imagery training comes from small pilot studies. In a series of studies with small samples of 6 to 8 patients in the experimental group Page and colleagues found improved upper arm function after combined physiotherapy and motor imagery training in chronic stroke patients [28,29] and in sub-acute patients (< 1 year post-stroke) [30]. A study by Stevens and Stoykov [31] reports a beneficial effect of motor imagery training in two stroke patients with an upper limb weakness. We also found a potential for motor imagery training in a preliminary study with chronic stoke patients [32]. Our study demonstrated enhanced performance in the experimental motor imagery group on the task that was practiced mentally, compared to the performance on this task in the group that did not engage in mental practice of movements, but used visual imagery. Finally, a study by Liu and colleagues [33] did not include detailed targeting of upper arm function but instead engaged patients in relearning broad and complex household tasks such as cooking and shopping using motor imagery. However, motor imagery training in these patients included, additional, learning of cognitive strategies such as task analysis and problem identification. Although these patients showed better relearning of motor tasks as a result of the training, they did not show improvement on motor function. The evidence so far indicates a potential for motor imagery as a rehabilitation technique, but mixed results as well as small sample sizes clearly warrants further investigation.

We hypothesise that patients engaging in mental rehearsal of their own movements (i.e. motor imagery) could show enhanced motor recovery through processes of functional redistribution of brain activity, as well as promoting perceived control which may be critical in good physical recovery after stroke. We intend to confirm the observations of the few preliminary studies suggesting that motor imagery training improves upper arm function in stroke survivors.

### Aims and objectives of the study

The aim of this study is to investigate the use of intensive motor imagery techniques in promoting recovery of arm function after stroke. The objective of this study is to establish the effectiveness of daily rehearsal of imagery of movements with the affected arm result in increasing functional recovery of that arm. It will also be established whether enhanced functional recovery of the arm due to motor imagery training is also associated with resultant increases in activities of daily living. It will be investigated whether the benefit of motor imagery training is related to individual differences in motor imagery ability in stroke patients. Similarly, it will be evaluated whether therapeutic benefit of motor imagery training is associated with either high perceived control at baseline, or an enhancement of perceived control of recovery as a result of motor imagery training.

## Methods/Design

### Study design

**Figure 1:**
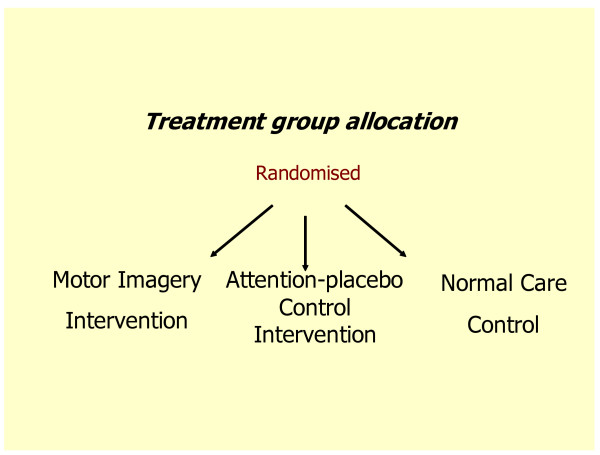
Study Design.

This is a three-arm randomised control trial with a single baseline and one outcome assessment. Patients are randomly assigned to one of three groups: the experimental patient group and two control patient groups (see Figure 1). The experimental group receives training in motor imagery (the motor imagery group). Patients in the first control group receive equally intensive training involving other forms of mental rehearsal that are not related to motor control, such as visual imagery of objects (attention-placebo control group). Patients in the second control group receive normal care with no additional training (normal care group). The attention-placebo control group is included to control for the effects of intensive training and therapist attention *per se*. Furthermore, the second control group receiving routine care only is needed in order to confirm that differences found between the experimental and placebo-control treatment indicate clinical benefit of motor imagery training rather than a decline in the attention-placebo control group. The experimental and attention-placebo control groups have the same duration of therapist contact and each therapist trains an equal number of patients in each group. Five weeks after baseline assessment all initial assessments related to outcome are repeated.

#### Ethical considerations

NHS Grampian and NHS Tayside ethical review boards governing each clinical centre have approved the study protocol REC Number 0310299. Written informed consent is obtained from each participant as set out by the local ethical review board. Clinical Trial Registration; current clinical trial protocol registration NCT00355836.

### The intervention

Patients engage in 45-minutes training per day during one-to-one sessions with a research therapist and further perform previously instructed tasks independently twice a week. Training is provided for four weeks.

The motor imagery training programme has been specifically developed to promote recovery of hand function through motor imagery by recruiting areas of the brain that could stimulate functional redistribution of brain activity [34-36]. This training programme focuses on the upper limb. Patients are asked to imagine upper limb movements and are trained to involve visual, kinaesthetic and combined images of movement. Patients are engaged in structured mental practice of a variety of elementary movements and activities of daily living. Motor imagery is facilitated through verbal instruction and feedback, observation of movement and visual display. Motor imagery is further evoked through movement illusion through the use of mirrors and video display.

The placebo-control training programme has been carefully devised to control effectively for the motor imagery intervention. This includes controlling for therapist attention as well as the cognitive functions indirectly recruited during the motor imagery that can account for the effect of motor imagery training. Therapist attention is controlled for by closely matching the intensity of the training to the experimental training programme as well as matching the belief in the effectiveness of the training that the researcher may convey to the patient. Cognitive demands that are controlled for include sustained attention, visualisation, memory demands, visual illusion, and inhibition.

The researchers providing the training closely follow a detailed operational manual to ensure that instructions, number of repetitions and the length of training are closely matched between patients and between the motor imagery and control training programmes.

### Eligibility

Patients are included according the following criteria: 1) a history of stroke one to six months prior to participation in the project, 2) Action Research Arm (ARA) test score of between 3 and 51 (maximum score 57; [37]) indicating a persistent motor weakness with the preserved ability to make some movement with the affected arm, 3) no severe aphasia (Token Test; [38]), 4) no alcohol dependency or evidence of substance abuse, 5) no severe cognitive impairment (Mental Status Questionnaire score of 7 or more; [39]).

### Recruitment

Patients are recruited in Scotland Grampian from the acute and chronic stroke units of Aberdeen Grampian University Hospitals NHS Trust, and furthermore in Scotland Tayside from Ninewells Hospital Tayside University Hospitals NHS Trust Dundee, from Perth Royal Infirmary and its partner rehabilitation locations. Consecutive recruitment is employed following CONSORT guidelines [40]. Consented patients enter the trial as soon as possible after the first month following stroke.

### Randomisation

Patients are randomly assigned to one of the three groups using a statistical minimising procedure [41]. Allocation is based on five stratification factors: age, sex, severity of motor impairment (baseline ARA test score), side of brain damage (left hemisphere, right hemisphere, or bilateral damage), and the time post-stroke. Statistical control variables include: the amount of other therapy (physio-, occupational-, and speech therapy) received during the training period, and the ability to perform motor imagery before and after intervention. The randomisation process is automated and happens following the baseline assessment.

### Blinding

All assessment is carried out by an examiner blind to the patient's group allocation.

### Power

Sample size is based on a power analysis. 135 patients will be recruited to complete the trial, allocating 45 patients to each of the three groups. The recruitment of 45 patients per group is aimed to achieve 80% power of detecting a difference of 0.6 standard deviations (SD) at the 5% significance level [42]. We suggest that a difference of 8 points on the scale would imply a meaningful difference and using an estimate of 12.5 for the standard deviation [43] relates to 0.6 SD. Previous studies that have examined the effect of intervention programmes using the same primary outcome measure (the Action Research Arm (ARA) test) have reported differences in excess of 8 units, which might be regarded as indicating a meaningful clinical benefit [44,45,30].

### Outcome

#### Primary and secondary outcome measures

##### Primary outcome measure

Action Research Arm (ARA) test [37], designed to assess recovery of the upper extremity function following cortical injury. The test includes four subscales: grasp, grip, pinch, and gross movement.

##### Secondary outcome measures

Grip strength (dynamometer; [46]), the standard nine hole pegboard task [47] and a newly developed peg board like task of timed manual dexterity performance, that is an integrated part of the motor imagery- and attention-placebo control training programmes. The latter task demonstrated the beneficial effect motor imagery training in a preliminary study of this work [32]. Activities of Daily Living (ADL) independence and level of functional limitations is assessed with the Barthel Index, BI [48], and the abridged Functional Limitation Profile (FLP; [49,50]) covering items of ambulation, body care, mobility, alertness, and communication.

#### Process variables

Motor imagery ability is assessed using a range of tasks. Individual differences between patients in motor imagery ability needs to be quantified, as this ability may vary depending on the lesion site [51] and hand function [52,53]. The following tasks have been developed to assess motor imagery ability, spontaneous motor imagery use, and the related issue of kinaesthetic working memory. 1) Mental chronometry: assessing the ability to predict, through mental imagery, the time necessary to perform movements [54]. 2) Motor imagery questions: adequate performance on this specially designed computer assisted reaction time task, depends on intact ability to mentally simulate movement in order to decide whether a statement is correct or not [55]. 3) Mental rotation of hands: adequate performance on this specially designed computer assisted reaction time task of left-right discrimination depends on intact ability to mentally simulate movement into the orientation of the stimulus [56]. 4) Kinaesthetic working memory: this newly developed working memory span procedure assesses the ability to reproduce movements through the kinaesthetic route. This ability is relevant in the evaluation of motor imagery ability and the beneficial effect of motor imagery training in stroke patients.

Perceived control is measured using the Recovery Locus of Control scale (RLOC; [21]). Emotional distress is assessed with the Hospital Anxiety and Depression Scale (HADS; [57]). A recovery self-efficacy questionnaire derived from Social Cognitive Theory [58] is included to assess patients' levels of perceived self-efficacy using the body care subscale from the FLP.

### Analysis

Analyses will be carried out to examine differences between the groups in the change in ARA test score from baseline to post-training assessment. Data will be analysed using analysis of covariance with pre-intervention assessment entered as a covariate. Assuming that pre-intervention assessment will be highly correlated to post-intervention assessment, this analysis will further increase the power of the study. Secondary outcome measures of hand function and process variables are analysed as above. Linear regression (with dummy variable coding of intervention group) are used to test whether any effect of intervention on ADL level, functional limitations, and perceived locus of control are explained by the targeted effect of hand function.

## Discussion

The idea of enhancing motor recovery through the use of motor imagery rehabilitation techniques is important with potential implications for clinical practice. The techniques evaluated as part of this randomised controlled trial are informed by the current understanding in cognitive neuroscience and the trial is both of scientific and applied interest.

## Competing interests

The author(s) declare that they have no competing interests.

## Authors' contributions

MI, MJ, HCD, RM and SH are grant holders, additional intellectual contributions were made by SJ and CS. All authors are site investigators who contributed to the development and writing of the protocol to its final version. All authors have been involved in the drafting and revision of this manuscript and have given approval of the final manuscript.

## Pre-publication history

The pre-publication history for this paper can be accessed here:



## References

[B1] Dijkerman HC, Wood VA, Langton Hewer R (1996). Long-term outcome after discharge from a stroke rehabilitation unit. J R Coll Physicians London.

[B2] Wade DT, Langton Hewer R (1987). Functional abilities after stroke: measurement, natural history and prognosis. J Neurol Neuros Psychi.

[B3] Feltz DL, Landers DM (1983). The effects of mental practice on motor skill learning and performance. A meta-analysis. J Sport Psych.

[B4] Driskell JE, Copper C, Moran A (1994). Does mental practice enhance performance?. J App Psych.

[B5] Martin KA, Moritz SE, Hall CR (1999). Imagery use in sports: A literature review and applied model. Sport Psychol.

[B6] Yue G, Cole KJ (1992). Strength increases from the motor program: comparison of training with maximal voluntary and imagined muscle contractions. J Neurophys.

[B7] Grouios G, Mousikou K, Hatzinikolaou K, Semoglou K, Kabitsis C (1997). The effect of a simulated mental practice technique on free throw shooting accuracy of highly skilled basketball players. J Hum Movem Studies.

[B8] Rogers RG (2006). Mental practice and acquisition of motor skills: examples from sports training and surgical education. Obstet Gyn Clin N Am.

[B9] Gentili R, Papaxanthis C, Pozzo T (2006). Improvement and generalization of arm motor performance through motor imagery practice. Neuroscience.

[B10] Weiller C (1995). Recovery from motor stroke: human positron emission tomopgraphy studies. Cereb Dis.

[B11] Marshall RS, Perera GM, Lazar RM, Krakauer JW, Constatine RC, DeLaPaz RL (2000). Evolution of cortical activation during recovery from corticospinal tract infaction. Stroke.

[B12] Decety J, Perani D, Jeannerod M, Bettinardi V, Tadary B, Woods R, Mazziotta JC, Fazio F (1994). Mapping motor representations with positron emission tomography. Nature.

[B13] de Lange FP, Hagoort P, Toni I (2005). Neural topography and content of movement representations. J Cog Neurosc.

[B14] Nudo RJ, Wise BM, SiFuentes F, Milliken GW (1996). Neural substrates for the effects of rehabilitative training on motor recovery after ischemic infarct. Science.

[B15] Nelles G, Jentzen W, Jueptnes M, Mueller S, Diener HC (2001). Arm training induced brain plasticity in stroke studied with serial Positron Emission Tomography. Neuroimage.

[B16] Carey JR, Kimberley TJ, Lewis SM, Auerbach EJ, Dorsey L, Rundquist P, Ugurbil K (2002). Analysis of fMRI and finger tracking training in subjects with chronic stroke. Brain.

[B17] Jang SH, Kim YH, Cho SH, Lee JH, Park JW, Kwon YH (2003). Cortical reorganization induced by task-oriented training in chronic hemiplegic stroke patients. Neuroreport.

[B18] Pascual-Leone A, Dang N, Cohen LG, Brasilneto JP, Cammarota A, Hallett M (1995). Modulation of muscle responses evoked by transcranial magnetic stimulation during the acquisition of new fine motor-skills. J Neurophys.

[B19] Jackson PL, Lafleur MF, Malouin F, Richards CL, Doyon J (2003). Functional cerebral reorganization following motor sequence learning through mental practice with motor imagery. Neuroimage.

[B20] Johnson-Frey SH (2004). Stimulation through simulation? Motor imagery and functional reorganization in hemiplegic stroke patients. Brain Cog.

[B21] Johnston M, Morrison V, MacWalter R, Partridge C (1999). Perceived control, coping and recovery from disability following stroke. PsycholHealth.

[B22] Partridge C, Johnston M (1989). Perceived control and recovery from stroke. Br J Clin Psych.

[B23] Robertson IH, Ridgeway V, Greenfield E, Parr A (1997). Motor recovery after stroke depends on intact sustained attention: a 2-year follow-up study. Neuropsychology.

[B24] Jackson PL, Lafleur MF, Malouin F, Richards C, Doyon J (2001). Potential role of mental practice using motor imagery in neurological rehabilitation. Arch Phys Med Rehab.

[B25] Lotze M, Halsband U (2006). Motor Imagery. J Physiology – Paris.

[B26] Sharma N, Pomeroy VM, Baron JC (2006). Motor Imagery – A backdoor to the motor system after stroke?. Stroke.

[B27] Braun SM, Beurskens AJ, Borm PJ, Schack T, Wade DT (2006). The effects of mental practice in stroke rehabilitation: A systematic review. Arch Phys Med Rehab.

[B28] Page SJ (2000). Imagery improves upper extremity motor function in chronic stroke patients: A pilot study. Occup Ther J Res.

[B29] Page SJ, Levine P, Leonard AC (2005). Effects of mental practice on affected limb use and function in chronic stroke. Arch Phys Med Rehab.

[B30] Page SJ, Levine P, Sisto S, Johnston MV (2001). A randomized efficiency and feasibility study of imagery in acute stroke. Clin Rehab.

[B31] Stevens JA, Phillips Stoykov ME (2003). Using motor imagery in the rehabilitation of hemiparesis. Arch Phys Med Rehab.

[B32] Dijkerman HC, Ietswaart M, Johnston, MacWalter RS (2004). Does motor imagery training improve hand function in chronic stroke patients? A pilot study. Clin Rehab.

[B33] Liu KP, Chan CC, Lee TM, Hui-Chan CW (2004). Mental imagery for promoting relearning for people after stroke: a randomized controlled trial. Arch Phys Med Rehab.

[B34] Annett J (1995). Motor imagery: perception or action?. Neuropsychologia.

[B35] Decety J, Grezes J (1999). Neural mechanisms subserving the perception of human actions. Trends Cogn Sci.

[B36] Decety J, Chaminade T (2003). When the self represents the other: A new cognitive neuroscience view on psychological identification. Conscious Cogn.

[B37] Lyle RC (1981). A performance test for assessment of upper limb function in physical rehabilitation treatment research. Int J Rehab Res.

[B38] De Renzi E, Faglioni P (1978). Normative data and screening power of a shortened version of the token test. Cortex.

[B39] Kahn RL, Goldfarb AI, Pollack KM, Peck A, Kane RA, Kane RL (1981). Mental status questionnaire [MSQ]. Assessing the elderly: A practical guide to measurement.

[B40] Moher D, Schulz KF, Altman DG (2001). The CONSORT statement: revised recommendations for improving the quality of reports of parallel group randomized trials. BMC Med Res Methodol.

[B41] Pocock ST (1983). Clinical Trials: A Practical Guide.

[B42] Machin D, Campbell M, Fayers P, Pinol A (1997). Sample size tables for clinical studies.

[B43] Van der Lee JH, De Groot V, Beckerman H, Wagenaar RC, Lankhorst GJ, Bouter LM (2001). The intra- and interrater reliability of the action research arm test: A practical test of upper extremity function in patients with stroke. Arch Phys Med Rehab.

[B44] Van der Lee JH, Wagenaar RC, Gustaaf J, Lankhorst GJ, Vogelaar TW, Devillé WL, Bouter LM (1999). Forced use of the upper extremity in chronic stroke patients. Stroke.

[B45] Dromerick AW, Edwards DF, Hahn M (2000). Does the application of constraint-induced movement therapy during acute rehabilitation reduce arm impairment after ischemic stroke?. Stroke.

[B46] Heller A, Wade DT, Wood VA, Sunderland A, Langton Hewer R, Ward E (1987). Arm function after stroke: measurement and recovery of the first three months. J Neurol Neuros Psychi.

[B47] Mathiowetz V, Weber K, Kashman N, Volland G (1983). Adult norms for the Nine Hole Peg Test of finger dexterity. Occup Ther J Res.

[B48] Mahony FL, Barthel DW (1965). Functional evaluation. The Barthel Index. Maryland State Med J.

[B49] Johnston M, Pollard B (1996). Report to NHS executive R&D programme for people with physical and complex disabilities.

[B50] Johnston M, Wright S, Weinman J (1995). Measures in Health Psychology: A Users Portfolio.

[B51] Sirigu A, Duhamel JR, Cohen L, Pillon B, Dubois B, Agid Y (1996). The mental representation of hand movements after parietal cortex damage. Science.

[B52] Parsons LM, Gabrieli JDE, Phelps EA, Gazzaniga MS (1998). Cerebrally lateralized mental representations of hand shape and movement. J Exp Psychol Human.

[B53] Nico D, Daprati E, Rigal F, Parsons L, Sirigu A (2004). Left and right hand recognition in upper limb amputees. Brain.

[B54] Sirigu A, Cohen L, Duhamel JR, Pillon B, Dubois B, Agid Y, Pierrot-Deseiligny C (1995). Congruent unilateral impairments for real and imagined hand movements. Neuroreport.

[B55] Goldenberg G, Podreka I, Steiner M, Willmes K, Suess E, Deecke L (1989). Regional cerebral blood-flow patterns in visual-imagery. Neuropsychologia.

[B56] Parsons LM (1994). Temporal and kinematic properties of motor behavior reflected in mentally simulated action. J Exp Psych-Human Perception And Performance.

[B57] Zigmund AS, Snaith RP (1983). Hospital Anxiety and Depression Scale. Acta Psychiatry Scand.

[B58] Bandura A (1997). Self-efficacy: the exercise of control.

